# Self-assessment of residents in respect of attitudes to communication

**DOI:** 10.1017/S1463423618000920

**Published:** 2019-04-12

**Authors:** Rustu Kaya, Ali Ramazan Benli, Aybala Cebecik, Didem Sunay

**Affiliations:** 1 Faculty of Health Sciences, Department of Healthcare Management, Yuksek Ihtisas University, Ankara, Turkey; 2 Faculty of Medicine, Department of Family Medicine, Karabuk University, Karabuk, Turkey

**Keywords:** communication skills, resident, self-assessment

## Abstract

**Aim:**

As communication skills are essential for medical practice, many medical schools have added communication skills training to their curricula in recent years. The aim of this study was to determine and compare the attitudes to communication skills of family medicine, internal medicine and general surgery residents.

**Materials and methods:**

Family medicine, internal medicine and general surgery residents of three training and research hospitals and one university hospital in Ankara were included in this cross-sectional study. A questionnaire was used for obtaining information about age, gender, marital status, graduation date and whether receiving any training for communication skills. The Turkish version of the Communication Skills Attitude Scale was used.

**Results:**

In all, 58 (50%) family medicine, 30 (25.9%) internal medicine, and 28 (24.1%) general surgery residents were accepted to participate in the study. Of the 116 residents, 58 (50%) were female and 58 (50%) were male, with a mean age of 29.47±4.63 years, and 68 (58.6%) of them were married; 59.5% of the participants received training about communication skills and 56.5% of them received it at medical school. The mean positive attitude scale (PAS) score was 3.85±0.58, and the mean negative attitude scale (NAS) score was 2.42±0.52. The PAS scores of female residents were higher than those of males (*P*=0.01). The PAS scores of residents who received communication skills training were higher than the scores of those who had not (*P*=0.01). The PAS scores of family medicine residents were higher and the NAS scores were lower than those of internal medicine and general surgery residents.

**Conclusion:**

The communication skill attitudes of family medicine residents were better than those of internal medicine and general surgery residents.

## Introduction

Interpersonal and communication skills are essential competencies for the practice of medicine. Effective communication strengthens the patient–physician relationship, improves compliance, patient satisfaction and the health outcomes in patients (Osorio *et al.*, 2012). Previous studies showed that patient–physician communication is a problem, the majority of errors in medical encounters arise from deficiencies in communication, and non-compliance of patients associated with lack of communication leads to a high annual cost to the national economy (Arda *et al*., 2014). Researchers have defined empathy skills of the healthcare provider as a key component in the professional–patient interaction for compliance with treatment, active participation of the patient in treatment and successful healthcare outcomes.

It is accepted that during training doctors need to be prepared for difficult situations such as the giving bad news, dealing with the development of drug resistance, specific situations causing ethical problems, dealing with difficult patients and sharing details and the diagnostic processes with patients’ relatives, in cases of life-threatening diseases (Sarıkaya *et al*., 2004). As communication is important and communication skills can be taught and improved, lessons in communication skills are included in the programs of medical faculties and in speciality training in developed countries. In Turkey, lesson on communication skills are recently included into the syllabus in medical faculty training, however, it is only included in the educational program of family physicians at the level of speciality training.

The communication skills of physicians and other healthcare professionals are known to affect patient satisfaction, treatment results and professional job satisfaction. In a previous study, some of the reasons for the dissatisfaction with healthcare services were demonstrated as communication problems such as newly qualified physicians not being prepared for the psychosocial concerns of the patient when taking the history, a low level of empathy and insufficient discussion of personal matters with the patient (Sarıkaya *et al*., 2004). The Accreditation Council for Graduate Medical Education (ACGME) requires that residents must be able to demonstrate interpersonal and communication skills that result in effective information exchange and collaboration with patients, patient families, and professional associates. The ACGME also requires residency programs to train residents in six competencies of patient care, system-based practice, interpersonal and communication skills, professionalism, medical knowledge, and practice-based learning and to develop evaluation methods to assess these competencies (Malik *et al*., 2012). In a study by Ishikawa *et al*. which evaluated the patient–physician relationship of residents, residents demonstrated relatively higher confidence in their communication skills in respect of gathering information and building the relationship, and they were seen to be less confident about sharing information and planning treatment (Ishikawa *et al*., 2014). We could not find any related study conducted in our country.

The aim of this study was to determine and compare the attitudes to communication skills of family medicine, internal medicine and general surgery residents.

## Materials and methods

This cross-sectional study included family medicine, internal medicine and general surgery residents of three training and research hospitals and one university hospital in Ankara between January 2015 and April 2015. A total of 184 residents were recruited for the study and of them 116 accepted to participate in the study. A questionnaire was used for obtaining information about age, gender, marital status, graduation date and whether receiving any training for communication skills. Turkish version of the Communication Skills Attitude Scale (CSAS) of Rees was used to assess communication skills (Rees and Garrud, 2001; Harlak *et al*., 2008). The scale was used in this study to evaluate the attitudes of residents, as self-ratings, self-reflection and self-monitoring are important for both medical faculty student and speciality students, which are essential for lifelong learning and improvement. The CSAS consists of 26 items within two subscales. Subscale I, the positive attitude scale (PAS), consists of 15 items (item nos. 1, 4, 5, 7, 8, 9, 10, 12, 13, 14, 16, 18, 21, 23 and 25) and subscale II, the negative attitude scale (NAS), consists of 11 items (item nos. 2, 3, 6, 11, 15, 17, 19, 20, 22, 24 and 26), in Turkish version and *α*s were 0.71 for NAS and 0.92 for PAS. All 26 items have choices along a 5-point Likert scale ranging from 1 (strongly disagree) to 5 (strongly agree). According to the results of the scale, PAS and NAS scores were calculated and recorded for each participant. Furthermore, some of the responses to the items in the scale were evaluated individually and used in comparisons among specialities. While deciding the statement to be evaluated individually, the items that can be assessed separate from scale are taken into account. The scores were also compared according to the gender and to the requirement of communication skill training. Approval for the study was granted by the ethics committee of Kecioren Training and Research Hospital (11.11.2015/996).

The data obtained in the study were evaluated using SPSS version 15.0. Descriptive statistics were stated as percentage and mean ± standard deviation (SD). Conformity of the data to normal distribution was assessed using the Kolmogorov-Smirnov test. In multiple comparisons of parametric data, the one-way ANOVA was used and *post hoc*, the Tukey B and Games-Howell tests were applied. In the analysis of non-parametric data, the chi-square test was used. A value of *P*<0.05 was accepted as statistically significant.

## Results

The study included 116/184 residents, as 58/114 in family medicine, 30/38 in internal medicine and 28/32 in general surgery departments. Of the residents, 58 (50%) were female and 58 (50%) were male, with a mean age of 29.47±4.63 years. Marital status was recorded as married by 68 (58.6%) residents, single by 47 (40.5%) and divorced by 1 (0.9%). The mean year of graduation was 2010±5.09 and the mean duration of residency was 2.05±1.10 years.

A total of 69 (59.5%) residents received communication skills training. Distribution of residents according to where communication skills training was received is shown in Table 1. No difference was determined between the specialities in terms of the status of having received communication skills training (*P*=0.218). When evaluation was made of where the communication skills training had been received according to specialities, it was seen that communication skills courses were reported to have been obtained during speciality training by 1 (5.88%) of 17 general surgery residents, by 3 (25%) of 12 internal medicine residents and by 18 (45%) of 40 family medicine residents.

**Table 1:** Distribution of residents according to where the communication skills training were received

	*n* (%)
Medical school	41 (59.4)
Specialist training	13 (18.8)
Medical school+specialist training	8 (11.6)
Focused courses and congresses	7 (10.1)

In the whole study group, the mean PAS score was 3.85±0.58, and the mean NAS score was 2.42±0.52. The PAS scores of female residents were higher than those of males (*P*=0.01) and no statistically significant difference was determined between the genders in respect of NAS scores (*P*=0.75). The PAS scores of residents who had taken a communication skills training were higher than the scores of those who had not obtained the training (*P*=0.01), and no statistically significant difference was determined in respect of NAS scores according to whether or not a communication skills training had been taken (*P*=0.19). When the PAS and NAS values were evaluated according to specialities, the PAS scores of family medicine residents were statistically significantly higher than those of internal medicine and general surgery residents (*P*=0.025 and *P*=0.020, respectively). No statistically significant difference was determined between the general surgery and internal medicine residents in respect of PAS scores (*P*=0.732). The NAS scores of family medicine residents were statistically significantly lower than those of internal medicine and general surgery residents (*P*=0.017 and *P*=0.0001, respectively). No statistically significant difference was determined between the general surgery and internal medicine residents in respect of NAS scores (*P*=0.534) (Table 2).

**Table 2:** PAS and NAS scores according to gender, communication skill education and specialities of residents

	PAS	NAS
*Gender*		
Female	3.96±0.44	2.34±0.54
Male	3.75±0.68	2.49±0.50
*P*	*0.01*	0.75
*Communication skills*		
Lessons taken	3.98±0.49	2.31±0.53
Lessons not taken	3.77±0.68	2.60±0.48
*P*	*0.01*	0.19
*Specialities*		
Family medicine	4.03±0.58	2.22±0.48
Internal medicine	3.74±0.42	2.56±0.55
Surgery	3.63±0.64	2.69±0.41
*P*		
Family – internal:	*0.025*	*0.017*
Family – surgery	*0.02*	*0.0001*
Internal – surgery	0.732	0.534

PAS=positive attitude scale; NAS=negative attitude scale.

Some of the responses to statements in the CSAS were evaluated individually. In response to the statement, 56.0% of the residents completely agreed, ‘*I must have good communication skills to be a good doctor*’, 55.2% completely disagreed to ‘*I don’t see any reason to learn communication skills*’, 43.1% agreed and 31.0% completely agreed to ‘*Developing communication skills is just as important as developing medical knowledge*’ 54.3% agreed and 32.8% completely agreed to ‘*I think that learning communication skills during medical training is very useful*’.

When comparisons of the responses to these statements were made according to the specialities of the residents, it was determined that in response to the item, ‘*I must have good communication skills to be a good doctor*’, there was no significant difference in respect of those who completely agreed between the family medicine and the internal medicine residents or between the general surgery and internal medicine residents (*P*=0.155, *P*=0.530, respectively), and a statistically significant higher rate of this response was given by family medicine residents compared to the general surgery residents (*P*=0.018).

Of those who completely disagreed with the item of ‘*I don’t see any reason to learn communication skills*’, no statistically significant difference was determined between family medicine and internal medicine residents (*P*=0.842) and a statistically significantly higher rate of this response was given by family medicine and internal medicine residents compared to the general surgery residents (*P*=0.001 and *P*=0.004, respectively).

In response to the item, ‘*Developing communication skills is just as important as developing medical knowledge*’ there was no significant difference in respect of those who completely agreed between the general surgery and internal medicine residents (*P*=0.967), and this response was given at a statistically significant higher rate by family medicine residents compared to the internal medicine and general surgery residents (*P*=0.009 and *P*=0.005, respectively).

In response to the item, ‘*I think that learning communication skills during medical training is very useful*’, the response of complete agreement was given at a statistically significant higher rate by family medicine residents compared to the internal medicine and general surgery residents (*P*=0.024 and *P*=0.030, respectively), and no statistically significant difference was determined between the internal medicine and general surgery residents (*P*=0.978).

When comparisons of the responses to these items were made according to gender, it was determined that in response to the items, ‘*I must have good communication skills to be a good doctor*’ and ‘*I think that learning communication skills during medical training is very useful*’, there was no significant difference in respect of gender of those who completely agreed (*P*=0.899 and *P*=0.378, respectively).

The responses of complete disagreement to the item, ‘*I don’t see any reason to learn communication skills*’, and complete agreement with the item, ‘*Developing communication skills is just as important as developing medical knowledge*’ were given at a statistically significantly higher rate by the female residents compared to the male residents (*P*=0.01 and *P*=0.02, respectively).

When comparisons of the responses to these items were made according to whether or not the participants had received communication skills lessons, the responses of complete agreement to the items, ‘*I must have good communication skills to be a good doctor*’, ‘*Developing communication skills is just as important as developing medical knowledge*’ and ‘*I think that learning communication skills during medical training is very useful*’, were determined to have been given at a statistically significantly higher rate by those who had received communication skills training compared to those who had not (*P*=0.017, *P*=0.008 and *P*=0.014, respectively). The response of complete disagreement to the item, ‘*I don’t see any reason to learn communication skills*’, was determined to have been given at a statistically significantly higher rate by those who had received communication skills training compared to those who had not (*P*=0.017 and *P*=0.004).

## Discussion

The results of the study that was aimed to determine and compare the attitudes to communication skills of family medicine, internal medicine and general surgery residents showed that in general the PAS points of all residents were high and the NAS points were low. The CSAS self-assessment evaluation scale was developed for medical faculty students by Rees *et al*. and was tested for validity and reliability in Turkish by Harlak *et al*. (Rees and Garrud, 2001) (Harlak *et al*., 2008).

The discussion on the concept of communication in healthcare was ongoing for the last 25–30 years, as a result of the need felt for communication and it has shown rapid development. That patient satisfaction is strongly affected by the communication skills of the physician has been reported in many studies (Elzubier, 2002). The communication skills of physicians and other healthcare personnel are known to affect patient satisfaction, treatment results and professional job satisfaction. In previous studies, some of the reasons for the dissatisfaction with healthcare services have been determined to be associated with communication problems (Elzubier, 2002).

In recent years, increased numbers of medical disputes and a crisis of confidence between doctors and patients have arisen from low competency (Zhu *et al*., 2016). Over the past decade, in Turkey there has been a significant deterioration in the relationship between patient and healthcare professionals. The physician–patient relationship presents an interpersonal process of the highest complexity. The definitive key for a healing patient–doctor relationship is professional communication (Street *et al*., 2009).

In a study by Arda *et al*., it was reported that 78.2% of physicians experienced communication problems with patients (Arda *et al*., 2014). In this context, it is recommended that communication skills, which are accepted as the basis of forming a patient–physician relationship, are included in medical education programs in a way that will support clinical knowledge and skills training. Accordingly, communication skills are currently included in the education programs of many medical faculties throughout the world (Perron *et al*., 2014).

However, such skills are still insufficiently addressed during internships and postgraduate training, despite the fact that they tend to decline unless regularly recalled and practised (Perron *et al*., 2014).

In the current study, 69/116 residents had received training in communication skills and most of them received it during medical education. While a statistically significant difference was determined in the PAS scores of those who had received communication skills training compared to those who had not, no significant difference was determined in the NAS scores. In addition, the responses of complete agreement to the items, ‘*I must have good communication skills to be a good doctor*’, ‘*Developing communication skills is just as important as developing medical knowledge*’ and ‘*I think that learning communication skills during medical training is very useful*’, were determined to have been given at a statistically significantly higher rate by those who had received communication skills training compared to those who had not. The response of complete disagreement to the item, ‘*I don’t see any reason to learn communication skills*’, was determined to have been given at a statistically significantly higher rate by those who had received communication skills training compared to those who had not. Rees and Garrud (2001) reported that the strongest factor in the correlation of negative attitudes towards communication skills learning was when students reported that their communication skills did not need improving.

When comparisons of the responses to these items were made according to gender, the PAS scores of female residents were found to be higher than those of male residents. The responses of complete disagreement to the item, ‘*I don’t see any reason to learn communication skills*’, and complete agreement with the item, ‘*Developing communication skills is just as important as developing medical knowledge*’ were given at a statistically significantly higher rate by female residents than male residents.

The results reported in studies that have examined communication skills of physicians according to gender were controversial. Some studies determined higher PAS scores in female medical faculty students (Malik *et al*., 2012; Busch *et al*., 2015). In a cohort study conducted in the UK, male medical faculty students were determined to have higher empathy scores compared to their female counterparts (Austin *et al*., 2007). A recent meta-analysis by Roter *et al*. (2002) indicated that female physicians are more likely to engage in patient-centred communication behaviours such as collaborative communication, empathetic communication and giving psychological information. In another study by Marteau *et al*. (1991), it was reported that male medical students were slower at learning communication skills compared to the female students.

McKinley *et al*. (2014) conducted a study to determine whether or not there was any difference between the genders of general surgery residents in respect of emotional intelligence. The results showed that females scored higher than males in the area of impulse control and relationships, and males scored higher than females in stress management and emotion management. When in general surgery, the female residents scored higher in impulse control, and males scored higher in stress management.

When the PAS and NAS scores were compared according to the specialities of the residents, the PAS scores of the family medicine residents were higher than those of the internal medicine and general surgery residents, and no significant difference was determined between the internal medicine and general surgery residents. When the NAS scores were examined, again the NAS points of the family medicine residents were determined to be lower than those of the other specialities, and no significant difference was determined between the internal medicine and general surgery residents. These results may be due to obtaining communication skills and patient-centred approach training during the speciality programme of family medicine.

Tariq *et al*. (2014) reported that most of the complaints about residents in university hospitals from nursing staff, other healthcare providers, patients and their families were related to attitude, communication and interpersonal skills; and in a study Zhu *et al* (2016) conducted on internal medicine residents, the overall communication scores were found to be moderate. In the same study, the residents also completed self-evaluations and it was concluded that residents underestimated their abilities compared to the results provided by other assessors. According to the results of current study, the PAS scores of residents were also moderate, but the results indicate that the residents were aware of the importance of communication for their profession.

In a study of general surgery residents by Zhu *et al*. (2016) the results indicated that residents should pay more attention to spending sufficient time with patients to be able to answer questions thoroughly. In comparison with other physicians, the general surgery residents were seen to be under greater pressure because their patients are at high risk with acute and severe illnesses and are therefore more prone to medical negligence and disputes. As well as the methodology of studies were different, according to the results of current study the communication skill scores of family medicine residents were better than general surgery residents. At the same time, a statistically significant higher rate of completely agreed response was given to the item ‘I *must have good communication skills to be a good doctor*’, by family medicine residents compared to the general surgery residents.

Family medicine residents were evaluated by patients using a communication assessment tool in a study by Myerholtz (2014) and the overall scores were found to be 67.0%. The scores of the first year residents were found to be higher than those of the second and third year residents, which the researchers attributed to the first year residents spending more time with the patients (Myerholtz, 2014). It was concluded that while it is plausible that the amount of time that a resident spends with the patient influences the patient’s perception of the resident’s communication skills. In a similar study by Myerholtz (2014), conducted 4 years later, the overall mean percentage of excellent scores was found to be 73%.

The education in most of the medical schools in Turkey is in the form of the biomedical model, which does not place much emphasis on communication skills during student training and evaluation (Harlak *et al*., 2008). In the biomedical model, the emphasis is principally on diseases and their management as opposed to managing the illness of an individual. There has been criticism of the biomedical approach as it is well known that not as much emphasis is placed on communication skills as in the bio-psychosocial model of care (Harlak *et al*., 2008).

The emergence of family medicine as a medical discipline is based on this principle. Family medicine is the only discipline that defines itself on the basis of relationships, and the core feature of the discipline in clinical applications is a patient-centred holistic approach rather than the disease (Kerr *et al*., 2011). Furthermore, there is an interview process with each specific patient, and with effective communication in that process, a relationship can be established between the doctor and the patient which can develop over time (Dikici *et al*., 2007). In the majority of medical schools in Turkey, basic communication skills training are given by the family medicine department.

The communication skills of a physician have a significant impact on patient health promotion and health education, especially in primary care practice where patients are first to come into contact with doctors (Alsaad *et al*., 2016).

Levinson and Roter (1995) demonstrated that the psychosocial attitudes of primary care physicians are related to their communication behaviours. Although high levels of psychosocial orientation in physicians and medical students are desirable, contemporary studies have shown a lack of increase or even a decline in empathy and patient centredness (Busch *et al*., 2015). Busch *et al*. (2015) concluded that the third year in medical education seems to be critical for psychosocial orientation since most medical curricula include more direct patient contact after year two. However, communication skills are still insufficiently addressed during internships and postgraduate training, despite the fact that they tend to decline unless regularly recalled and practised (Osorio *et al*., 2012). Perhaps the most significant limitation of the study was the failure to include all speciality residents and all training hospitals in the city.

Another limitation was, in the current study, the residents completed self-assessment evaluations. Many different methods have been applied to assess the communication skills of medical students and residents, including a behavioural checklist for direct or video observation, objective structured clinical examinations and patient satisfaction surveys. No single evaluation method will be able to truly capture a physician’s competence in the complex behaviours that constitute interpersonal and communication skills (Brennan *et al*., 2010). Self-assessment has been identified as a vital aspect of professional self-regulation. However, evidence from a large systematic review of the accuracy of physician self-assessment suggested that physicians have a limited ability to accurately self-assess (Tariq *et al*., 2014). Low response rate from family medicine compared to other disciplines and small sample size were the other limitations of the study that restricts the possibility of generalising the results.

In conclusion, even though self-assessments were made, the results of this study showed that there is a need for residents, particularly general surgery department residents, to have an increased awareness of communication skills. These findings also indicate that considerable effort may be needed to initiate a change in resident attitudes about communication skills.

## Conflict of Interest

The authors declare that there is no conflict of interest regarding the publication of this paper.
